# *Achillea erba-rotta* subsp. *moschata* (Wulfen) I. Richardson Modulates Inflammatory and Antioxidant Pathways in Brain Endothelial and Microglial Cells

**DOI:** 10.3390/ph19060832

**Published:** 2026-05-27

**Authors:** Benedetta Mercuriali, Martina Bottoni, Fabrizia Milani, Majeda Muluhie, Laura Santagostini, Claudia Giuliani, Joanna Rzemieniec, Laura Castiglioni, Gelsomina Fico, Luigi Sironi

**Affiliations:** 1 Laboratory of Pharmacology of Thrombosis and Atherosclerosis, Department of Pharmaceutical Sciences, University of Milan, Via Luigi Mangiagalli, 25, 20133 Milan, Italy; majeda.muluhie@unimi.it (M.M.); joanna.rzemieniec@unimi.it (J.R.); laura.castiglioni@unimi.it (L.C.); luigi.sironi@unimi.it (L.S.); 2 Botanical Garden G.E. Ghirardi, Department of Pharmaceutical Sciences, University of Milan, Via Religione 25, 25088 Toscolano Maderno, Italy; martina.bottoni@unimi.it (M.B.); fabrizia.milani@unimi.it (F.M.); claudia.giuliani@unimi.it (C.G.); gelsomina.fico@unimi.it (G.F.); 3 Department of Chemistry, University of Milan, 20133 Milan, Italy; laura.santagostini@unimi.it; 4 Laboratory of Plant Biology, Department of Pharmaceutical Sciences, University of Milan, 20133 Milan, Italy

**Keywords:** *Achillea erba-rotta* subsp. *moschata* (Wulfen) I. Richardson, microglia, blood–brain barrier, neuroinflammation, neuropharmacology, anti-inflammatory, antioxidant, aryl hydrocarbon receptor

## Abstract

**Background**. Neuroinflammation, driven by chronic microglial activation and blood–brain barrier dysfunction, is increasingly recognised as a key pathogenic mechanism in neurodegenerative disorders. *Achillea erba-rotta* subsp. *moschata*, an alpine medicinal plant traditionally employed for inflammatory conditions, has demonstrated antioxidant and anti-inflammatory properties; however, its effects on brain-related cell types and the underlying neuropharmacological mechanisms remain largely unexplored. This study investigated the molecular pathways by which *A. erba-rotta* subsp. *moschata* modulates neuroinflammation in key cellular components of the neurovascular unit. **Methods**. We evaluated the pharmacological activity of *A. erba-rotta* subsp. *moschata* aqueous extract (20–200 µg/mL) in LPS-stimulated BV2 microglial cells and human brain microvascular endothelial cells (hBMECs). Molecular mechanisms were characterised using qRT-PCR and Western blot analysis, focusing on inflammatory, antioxidant (Nrf2/HO-1), and AhR signalling pathways. **Results**. *A. erba-rotta* subsp. *moschata* extract significantly attenuates inflammatory responses in both cell types. In BV2 microglia, the extract reduced pro-inflammatory mediators and promoted anti-inflammatory signalling, including dose-dependent upregulation of TGF-β. In parallel, in hBMECs, the extract preserved endothelial integrity and mitigated inflammation-induced alterations without affecting cell viability. At the molecular level, the extract modulated key transcriptional pathways involved in inflammation and redox homeostasis, including NF-κB and the Nrf2/HO-1 axis. Importantly, robust CYP1A1 induction indicated aryl hydrocarbon receptor (AhR) activation, revealing coordinated crosstalk between inflammatory and antioxidant pathways. **Conclusions**. *A. erba-rotta* subsp. *moschata* exerts balanced, tissue-dependent immunomodulatory activity through multi-target neuropharmacological mechanisms. The anti-inflammatory effects in microglia combined with barrier-preserving actions in brain endothelium, support its therapeutic potential as a neuropharmacological agent for neuroinflammatory disorders.

## 1. Introduction

Neuroinflammation is increasingly recognised as a pivotal process in the pathogenesis of various neurological disorders, including Alzheimer’s disease, Parkinson’s disease and stroke-induced ischaemia [1,2]. This inflammatory response is primarily mediated by the activation of microglia, the resident macrophage-like cells of the central nervous system [3]. While microglial cells are essential for host defence and tissue repair in the brain, their overactivation leads to the excessive production and accumulation of pro-inflammatory and cytotoxic substances such as nitric oxide (NO), various cytokines, and prostaglandins, which are directly deleterious to neurons and to blood–brain barrier (BBB) integrity [4,5,6]. Furthermore, this chronic inflammatory state can exacerbate oxidative stress, creating a vicious cycle that profoundly contributes to neurodegeneration. In this context, natural compounds have gained attention for their potential to modulate neuroinflammatory processes through multiple complementary mechanisms. Beyond their well-documented antioxidant properties, many bioactive compounds also exert direct anti-inflammatory effects on signalling pathways [7,8,9].

Phytochemicals such as flavonoids, terpenes, and phenolic acids are known to regulate signalling cascades including NF-κB, MAPK, and Nrf2, thereby attenuating the production of nitric oxide and pro-inflammatory mediators [10,11,12,13]. Within this context, extensive studies have highlighted apigenin and luteolin’s neuroprotective and neuro-immunomodulatory capabilities in various neuroinflammation models pertinent to Alzheimer’s disease and other neurodegenerative diseases [14,15]. The mechanisms of action of these flavonoids include inhibiting the production of NO in microglia, attenuating microglial activation and reducing the overproduction of pro-inflammatory cytokines such as TNF-α, IL-1β, and IL-6 [16].

In this context, the Achillea genus, belonging to the Asteraceae family, represents a valuable source of bioactive metabolites [17]. Historically, Achillea species have been extensively used in traditional medicine across diverse cultures for their recognised medicinal properties, including anti-inflammatory, wound-healing, and gastrointestinal benefits. Several species, including *A. millefolium* and *A. ligustica*, have demonstrated anti-inflammatory and antioxidant properties linked to their content of flavonoids (e.g., apigenin, luteolin) and phenolic acids [18,19].

*Achillea erba-rotta* subsp. *moschata* (Wulfen) I. Richardson (syn. *Achillea moschata* Wulfen [20]), an endemic alpine plant, is also widely recognised in traditional practices [21,22,23]. Recent phytochemical characterisation of its traditional aqueous preparation by LC-MS/MS analysis revealed bioactive compounds including phenolic acids (caffeoylquinic derivatives), flavones (apigenin, luteolin), and flavonols (quercetin, kaempferol, isorhamnetin) [24,25,26]. Recent data demonstrated Nrf2 activation and anti-inflammatory activity in peripheral cell models [24,25]. However, critical knowledge gaps remain regarding its neuropharmacological mechanisms in brain cell models. Specifically, while apigenin and luteolin are known NF-κB and Nrf2 modulators in isolated form [14,15], whether the complex phytochemical mixture in *A. erba-rotta* extract engages these pathways in brain endothelial and microglial cells has not been established. Moreover, potential crosstalk between Nrf2 antioxidant signalling and the aryl hydrocarbon receptor (AhR) pathway, increasingly recognised as a regulator of neuroinflammatory responses, has not been investigated for any Achillea species [27,28,29,30,31]. Finally, effects on blood–brain barrier integrity, a critical determinant of neuroinflammatory progression, remain uncharacterised.

We hypothesised that the traditional *A. erba-rotta* subsp. *moschata* preparation would modulate multiple neuroprotective pathways in brain cells, including inflammatory mediator suppression, Nrf2-dependent antioxidant activation, engagement of the AhR-CYP1A1 axis (a novel mechanism for Achillea species), and preservation of blood–brain barrier tight junction integrity. Using human brain microvascular endothelial cells and murine microglia, we tested this hypothesis through a combination of gene expression and protein-level pathway analyses.

## 2. Results

### 2.1. Effect of A. erba-rotta subsp. moschata Extract on XTT Assay in hBMEC and BV2 Cells

**Figure 1:**
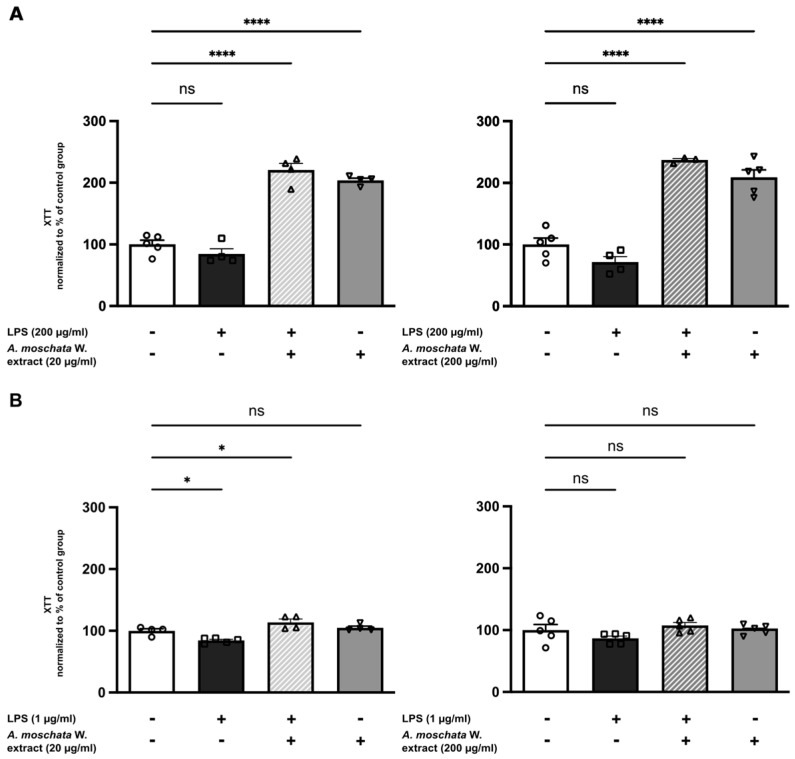
*Achillea erba-rotta* subsp. *moschata* increases cell viability. Cell viability analysis of hBMECs (**A**) and BV2 (**B**) cells assessed by XTT assay after 6 h treatment with *A. erba−rotta* subsp. *moschata* extract at different concentrations, with or without LPS stimulation. Data are shown as mean ± SD (n = 5–6/each group), untreated control group set = 100%. For statistical analysis, one-way ANOVA and post hoc Holm–Sidak’s multiple comparisons test were used. * *p* < 0.05 and **** *p* < 0.0001 vs. control untreated group; ns, not significant.

To assess potential cytotoxic effects, the impact of *A. erba-rotta* subsp. *moschata* extract was evaluated in hBMEC (Figure 1A) and BV2 cells (Figure 1B) using the XTT viability assay. Treatment with the extract produced a significant increase in XTT absorbance compared with untreated controls. This effect was evident in both cell types, under basal conditions as well as following LPS stimulation (Figure 1).

### 2.2. Effects of A. erba-rotta subsp. moschata Extract in Human Brain Microvascular Endothelial Cells

#### 2.2.1. Modulation of Inflammatory Mediators and Adhesion Molecules

**Figure 2:**
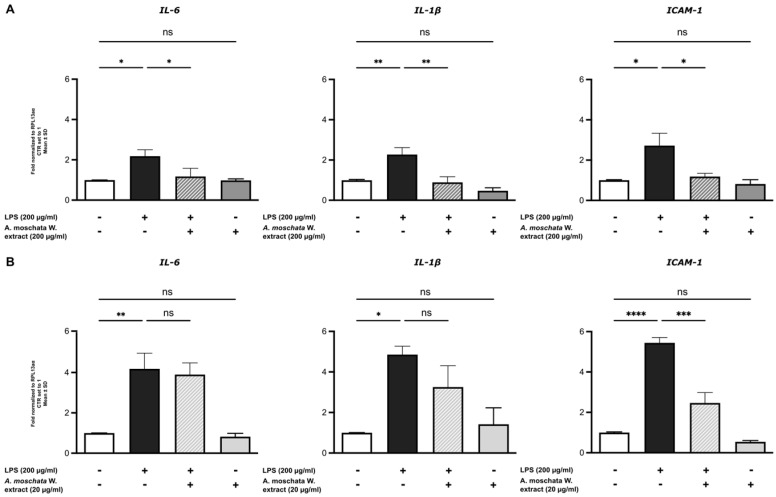
*Achillea erba-rotta* subsp. *moschata* modulates inflammation in hBMECs. Quantitative analysis of pro-inflammatory markers in hBMECs treated with *A. erba-rotta* subsp. *moschata* extract for 6 h at 200 μg/mL (**A**) and 20 μg/mL (**B**) with or without LPS stimulation. Graphs show mRNA expression of interleukin-6 (IL-6), interleukin-1β (IL-1β) and intercellular adhesion molecule-1 (ICAM-1), normalised to RPL13α. Data are shown as mean ± SD (n = 3 independent experiments), untreated control group set = 1. For statistical analysis, one-way ANOVA and post hoc Holm–Sidak’s multiple comparisons test were used. * *p* < 0.05, ** *p* < 0.01, *** *p* < 0.001 and **** *p* < 0.0001 vs. LPS group; ns, not significant.

To elucidate the potential mechanisms underlying the anti-inflammatory effects of *A. erba-rotta* subsp. *moschata* extract, we investigated its impact on inflammatory cytokine production. LPS stimulation significantly increased the mRNA expression of pro-inflammatory cytokines interleukin-6 (IL-6) and interleukin-1β (IL-1β), as well as the expression of the adhesion molecule intercellular adhesion molecule-1 (ICAM-1). Treatment with *A. erba-rotta* subsp. *moschata* at the higher concentration (200 μg/mL) significantly diminished the LPS-induced expression of IL-6 (*p* < 0.05), IL-1β (*p* < 0.01) and ICAM-1 (*p* < 0.05) (Figure 2A). At the lower concentration (20 μg/mL), the extract significantly suppressed ICAM-1 expression (*p* < 0.001), while effects on IL-6 and IL-1β did not reach statistical significance (Figure 2B). Overall, these findings demonstrate that *A. erba-rotta* subsp. *moschata* exerts dose-dependent modulatory effects on inflammatory responses in hBMECs, contributing to the regulation of soluble mediators and cellular adhesion molecules.

Importantly, treatment with *A. erba-rotta* subsp. *moschata* alone did not cause detectable alterations compared to untreated controls, indicating the absence of intrinsic pro-inflammatory activity.

#### 2.2.2. Induction of AhR-Pathway Following *A. erba-rotta* subsp. *moschata* Extract Treatment

**Figure 3:**
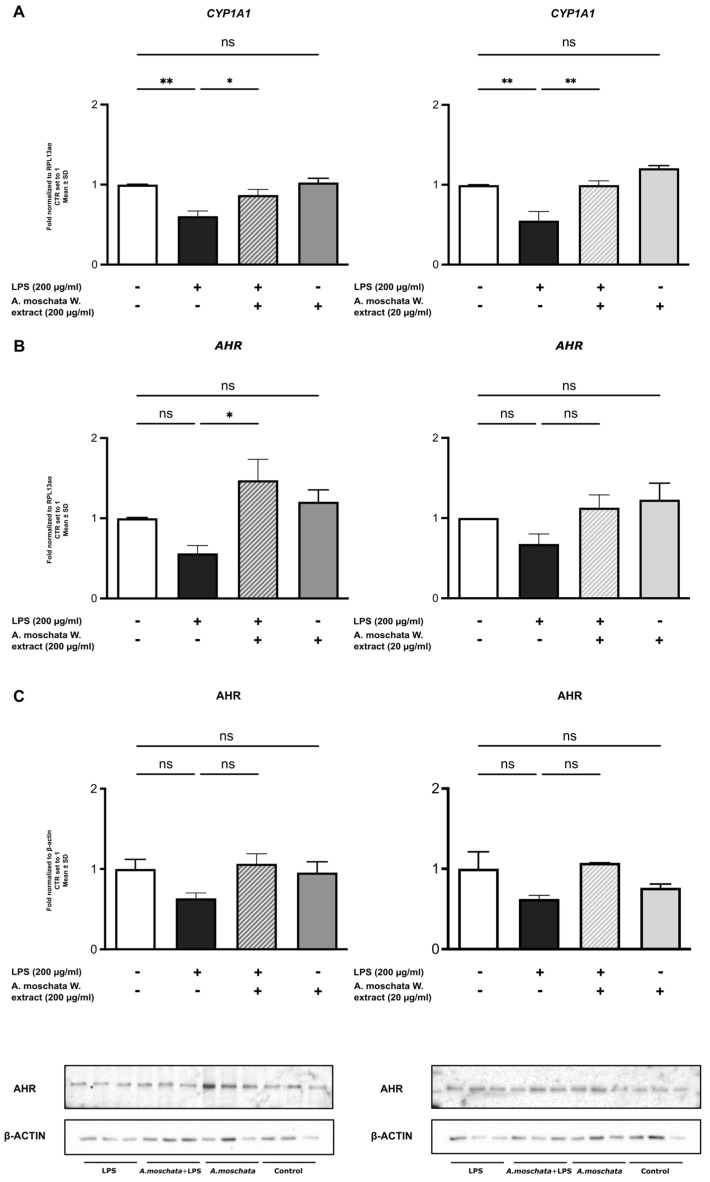
*A. erba-rotta* subsp. *moschata* modulates AhR–CYP1A1 axis in hBMECs. Modulation of the aryl hydrocarbon receptor (AhR) signalling pathway in hBMEC. Cells were treated with *A. erba-rotta* subsp. *moschata* extract for 6 h, at two different concentrations (20 and 200 μg/mL), with or without LPS stimulation. Quantitative analysis of CYP1A1 (**A**) and AHR (**B**). Their expressions were normalised on RPL13α. Protein levels of AHR (**C**) were evaluated by Western blot analysis. Densitometric quantification was performed and normalised to β-actin. Data are shown as mean ± SD (n = 3 independent experiments), untreated control group set = 1. For statistical analysis, one-way ANOVA and post hoc Holm–Sidak’s multiple comparisons test were used. * *p* < 0.05 and ** *p* < 0.01 vs. LPS group; ns, not significant.

Different natural bioactive compounds have been seen to modulate the aryl hydrocarbon receptor (AhR), a transcription factor that plays a role in xenobiotic metabolism and immune regulation, through modulation of inflammatory responses by cytochrome P450 1A1 (CYP1A1). In our study, LPS stimulation significantly reduced CYP1A1 mRNA levels. Treatment with *A. erba-rotta* subsp. moschata, at both tested concentrations, effectively restored CYP1A1 expression (*p* < 0.01; Figure 3A). Given the role of CYP1A1 as a target gene of AhR, we further assessed AhR expression at both mRNA and protein levels. LPS stimulation decreased AhR expression, and treatment with the extract at 200 μg/mL significantly increased AhR mRNA levels (*p* < 0.05; Figure 3B). Protein-level analysis confirmed modulation of AhR expression (Figure 3C). These results suggest that *A. erba-rotta* subsp. *moschata* phytochemicals can engage the AhR–CYP1A1 signalling axis, thereby counteracting LPS-induced dysregulation. This represents a potential mechanism through which the extract may exert protective effects against inflammation and oxidative stress. Importantly, *A. erba-rotta* subsp. *moschata* alone did not produce detectable alterations compared with untreated controls, indicating context-dependent effects.

#### 2.2.3. *A. erba-rotta* subsp. *moschata* Modulates Tight Junctions’ Expression Pathways

**Figure 4:**
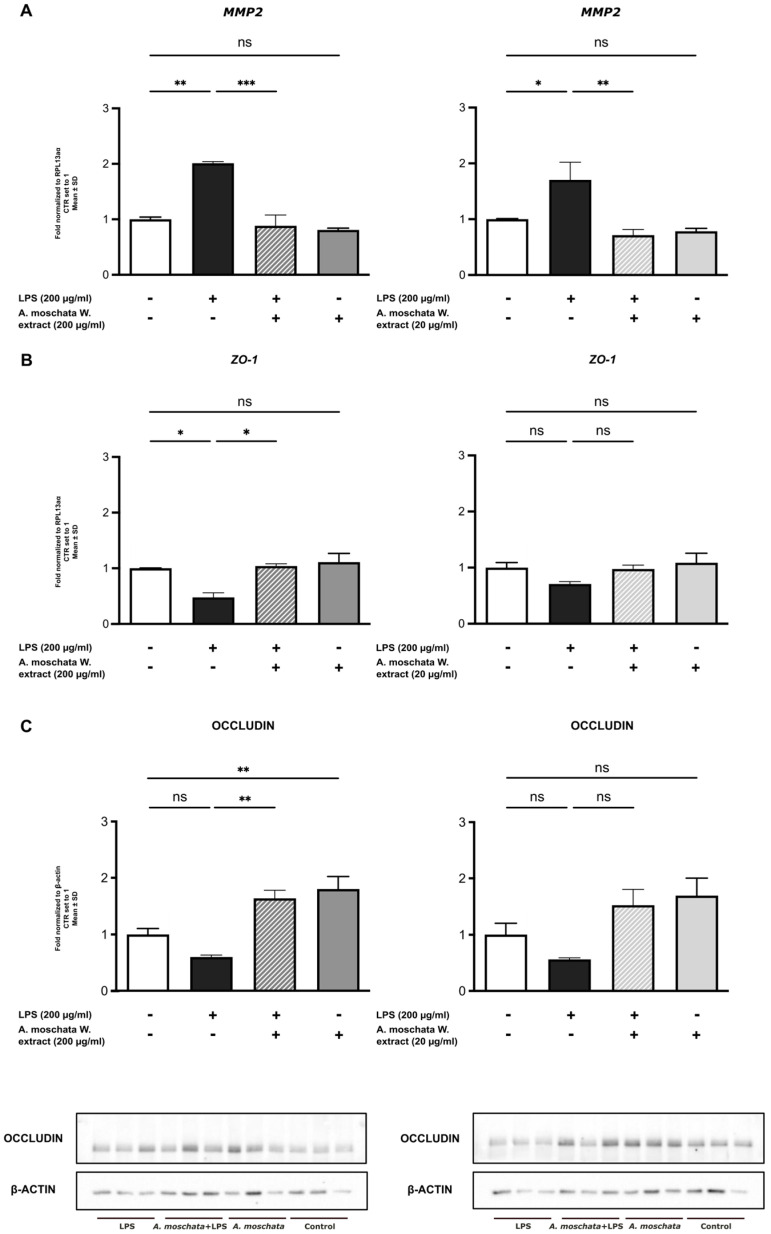
*Achillea erba-rotta* subsp. *moschata* attenuates tight junction degradation in hBMECs. Analysis of tight junction-associated proteins in hBMEC treated with *A. erba−rotta* subsp. *moschata* extract for 6 h, at two different concentrations (20 and 200 μg/mL), with or without LPS stimulation. Quantitative analysis of MMP2 (**A**) and ZO−1 (**B**). Their expressions were normalised on RPL13α. Protein levels of occludin (**C**) were evaluated by Western blot analysis. Densitometric quantification was performed and normalised to β−actin. Data are shown as mean ± SD (n = 3 independent experiments), untreated control group set = 1. For statistical analysis, one-way ANOVA and post hoc Holm−Sidak’s multiple comparisons test were used. * *p* < 0.05, ** *p* < 0.01 and *** *p* < 0.001 vs. LPS group; ns, not significant.

Tight junctions, a key structural and functional component of the blood–brain barrier (BBB), ensure selective permeability and the maintenance of central nervous system homeostasis. LPS stimulation significantly upregulated matrix metallopeptidase-2 (MMP2) gene expression in hBMECs. Treatment with *A. erba-rotta* subsp. *moschata* extract at both concentrations restored MMP2 expression to control levels (200 µg/mL: *p* < 0.01; 20 µg/mL: *p* < 0.001 vs. LPS; Figure 4A). LPS treatment also reduced zonula occludens-1 (ZO-1) gene expression, and this decrease was counteracted by *A. erba-rotta* subsp. *moschata* at the higher concentration (*p* < 0.05; Figure 4B). At the protein level, while LPS did not significantly reduce occludin expression, treatment with *A. erba-rotta* subsp. *moschata* at 200 µg/mL significantly increased occludin level compared to LPS-treated cells (*p* < 0.01; Figure 4C). These findings suggest that *A. erba-rotta* subsp. *moschata* contributes to the preservation of BBB integrity by modulating tight junction associated molecules, suggesting a protective role in maintaining barrier functionality under inflammatory conditions.

Notably, treatment with *A. erba-rotta* subsp. *moschata* alone did not produce detectable alterations compared with untreated controls, indicating context-dependent effects.

### 2.3. Effects of A. erba-rotta subsp. moschata Extract in Murine Microglial Cells

#### 2.3.1. Reduction in Pro-Inflammatory Cytokines

**Figure 5:**
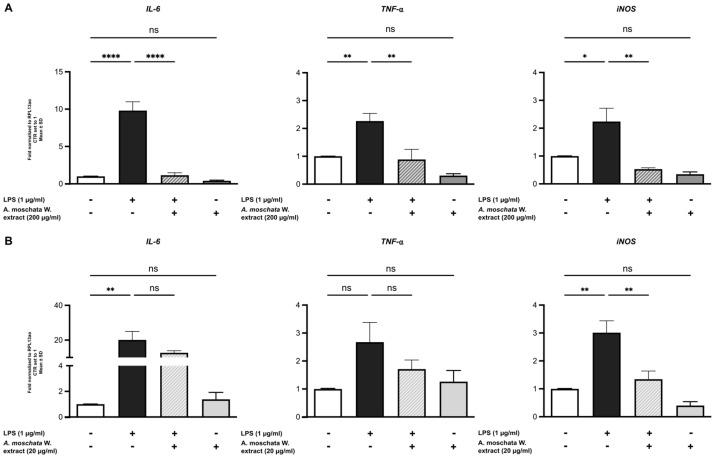
*Achillea erba-rotta* subsp. *moschata* suppresses inflammatory mediators in BV2 microglial cells. Quantitative analysis of pro-inflammatory markers in murine microglial cells BV2 treated with *A. erba−rotta* subsp. *moschata* extract for 6 h, at 200 μg/mL (**A**) and 20 μg/mL (**B**), with or without LPS stimulation. Graphs show mRNA expression of interleukin-6 (IL-6), tumour necrosis factor-α (TNF−α) and inducible nitric oxide synthase (iNOS), normalised to RPL13α. Data are shown as mean ± SD (n = 3 independent experiments), untreated control group set = 1. For statistical analysis, one-way ANOVA and post hoc Holm–Sidak’s multiple comparisons test were used. * *p* < 0.05, ** *p* < 0.01 and **** *p* < 0.0001 vs. LPS group; ns, not significant.

To obtain a more comprehensive overview of the neuroinflammatory process, we evaluated the expression of inflammatory cytokines in BV2 microglial cells. LPS stimulation significantly increased the levels of the pro-inflammatory cytokines IL-6 and TNF-α, as well as the expression of inducible nitric oxide synthase (iNOS), compared to untreated control cells (Figure 5A). Treatment with *A. erba-rotta* subsp. *moschata* at higher concentration (200 μg/mL) significantly reduced the LPS-induced expression of IL-6 (*p* < 0.0001), TNF-α (*p* < 0.01) and iNOS (*p* < 0.05; Figure 5A). At the lower concentration (20 μg/mL), the extract significantly suppressed iNOS expression (*p* < 0.01), while effects of IL-6 and TNF-α did not reach statistical significance (Figure 5B). These findings indicate that *A. erba-rotta* subsp. *moschata* exerts a dose-dependent protective effect by attenuating key mediators of neuroinflammation in microglial cells.

Importantly, treatment with *A. erba-rotta* subsp. *moschata* alone, at either concentration, did not cause detectable alterations compared with untreated controls.

#### 2.3.2. *A. erba-rotta* subsp. *moschata* Improves Antioxidant Pathway

Given the high flavonoid content of *A. erba-rotta* subsp. *moschata*, which is known to influence cellular antioxidant defence mechanisms, we focused our analysis on the nuclear factor erythroid 2–related factor 2 (Nrf2). Nrf2 acts as a central regulator of the antioxidant response by controlling the expression of cytoprotective genes, including heme oxygenase-1 (HO-1). This pathway plays a crucial role in mitigating oxidative stress and limiting the damage associated with inflammation.

**Figure 6:**
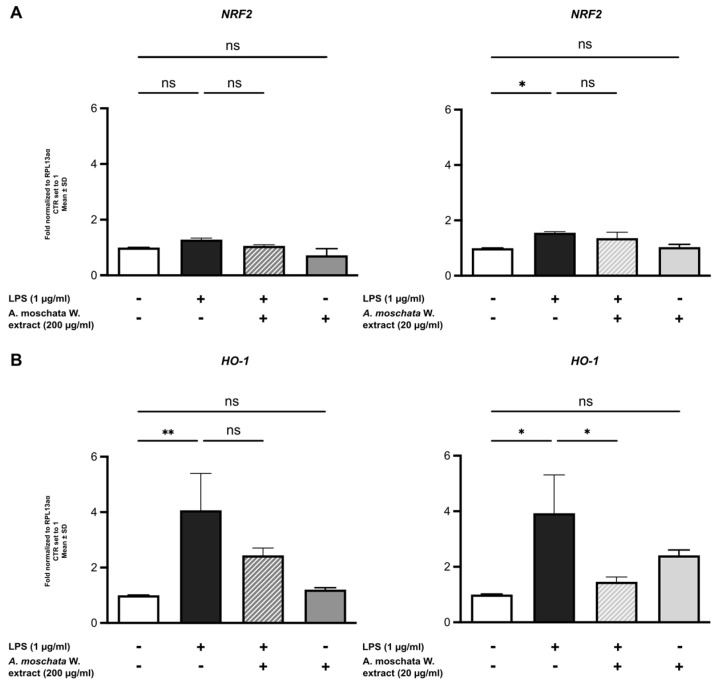
*A. erba-rotta* subsp. *moschata* changes in antioxidant pathway in BV2 cells. Quantitative analysis of antioxidant pathway components in murine microglial cells BV2 treated with *A. erba-rotta* subsp. *moschata* extract for 6 h, at two different concentrations (20 and 200 μg/mL), with or without LPS stimulation. Graphs show mRNA expression of NRF2 (**A**) and HO-1 (**B**), normalised to RPL13α. Data are shown as mean ± SD (n = 3 independent experiments), untreated control group set = 1. For statistical analysis, one-way ANOVA and post hoc Holm–Sidak’s multiple comparisons test were used. * *p* < 0.05 and ** *p* < 0.01 vs. LPS group; ns, not significant.

The results demonstrate that LPS treatment upregulates Nrf2 gene expression, indicating activation of the antioxidant response pathway during inflammatory stimulation (Figure 6A). Interestingly, treatment with *A. erba-rotta* subsp. *moschata* did not change Nrf2 levels, compared to LPS-treated cells.

Analysis of HO-1 mRNA, a key downstream target of Nrf2, showed that LPS treatment significantly increased HO-1 expression. Treatment with *A. erba-rotta* subsp. *moschata* at 200 μg/mL maintained the LPS-induced HO-1 upregulation, while treatment at 20 μg/mL inhibited LPS-induced HO-1 expression (*p* < 0.05; Figure 6B). These findings suggest that *A. erba-rotta* subsp. *moschata* extract does not suppress the Nrf2/HO-1 antioxidant response activated during inflammatory stimuli. Under inflammatory conditions, cells activate this pathway as an endogenous defence mechanism against oxidative stress, and the extract at 200 μg/mL supports the maintenance of this protective cellular response.

#### 2.3.3. Upregulation of TGF-β Expression

**Figure 7:**
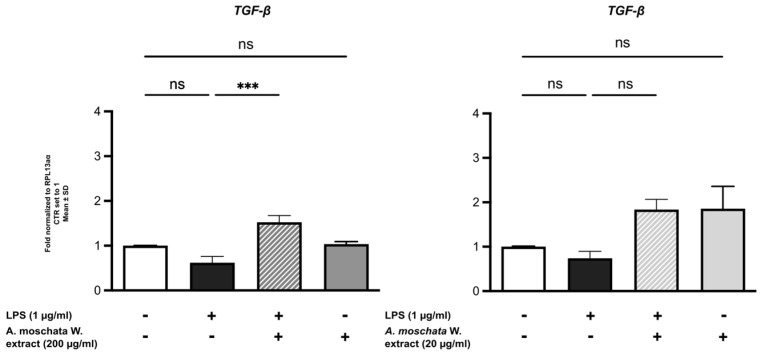
Immunomodulation in BV2 after *A. erba-rotta* subsp. *moschata* treatment. Quantitative analysis of transforming growth factor-β (TGF−β) in murine microglia cells BV2 treated with *A. erba-rotta* subsp. *moschata* extract for 6 h, at two different concentrations (20 and 200 μg/mL), with or without LPS stimulation. Graphs show mRNA expression of TGF-β, normalised to RPL13α. Data are shown as mean ± SD (n = 3 independent experiments), untreated control group set = 1. For statistical analysis, one-way ANOVA and post hoc Holm–Sidak’s multiple comparisons test were used. *** *p* < 0.001 vs. LPS group; ns, not significant.

Finally, we examined the impact of *A. erba-rotta* subsp. *moschata* on the immunomodulatory activity of microglial cells, a process known to be influenced by the plant’s flavonoid-rich composition. LPS treatment alone shows a non-significant trend toward decreased transforming growth factor-β (TGF-β) expression. Treatment with *A. erba-rotta* subsp. *moschata* in the presence of LPS resulted in increased TGF-β levels, with this effect reaching statistical significance at 200 μg/mL (*p* < 0.001; Figure 7). This upregulation of TGF-β expression suggests that *A. erba-rotta* subsp. *moschata* extract may facilitate the transition from acute inflammatory responses to resolution and tissue homeostasis.

## 3. Discussion

Neuroinflammation represents a critical pathophysiological mechanism underlying numerous neurodegenerative disorders, where endothelial dysfunction at the blood–brain barrier (BBB) and chronic activation of microglia contribute to progressive neuronal damage [1,2]. While previous studies have characterised the phytochemical composition of *A. erba-rotta* subsp. *moschata* and demonstrated anti-inflammatory and antioxidant properties in peripheral cellular models, particularly gastrointestinal epithelial cells [25,26], the specific effects on brain cell types and the underlying molecular mechanisms in neuroinflammatory contexts remain unexplored. This study provides the first characterisation of *A. erba-rotta* subsp. *moschata* effects in brain cellular models, demonstrating modulation of inflammatory pathway, preservation of BBB integrity, engagement of the AhR-CYP1A1 axis, and maintenance of antioxidant responses in both human brain microvascular endothelial cells and murine microglial cells.

The XTT viability assay demonstrated increased absorbance following extract treatment in both cell types. While this may indicate enhanced cellular metabolic activity, potential direct reduction in the tetrazolium salt by antioxidant phytochemicals cannot be excluded. Given that *A. erba-rotta* subsp. *moschata* contains flavonoids and phenolic acids, compounds with well-documented antioxidant and reducing activities, the observed increase may reflect either cellular effects, chemical interference, or both.

At concentrations consistent with traditional use, the extract modulates inflammatory mediator expression in both microglial and endothelial cells [20,21,22,24]. In BV2 microglial cells, treatment at the concentration of 200 µg/mL significantly reduced LPS-induced expression of IL-6, TNF-α, and iNOS. The downregulation of iNOS is particularly relevant as it indicates reduced nitric oxide production, a reactive nitrogen species that contributes to oxidative stress and neuronal damage during neuroinflammation [32,33,34]. Similarly, Elmann and collaborators [17] reported a reduction in LPS-induced inflammatory mediators in primary microglial cultures treated with Achillea fragrantissima, supporting the relevance of the experimental model. In hBMECs, the extract reduced IL-6, IL-1β, and notably ICAM-1, an adhesion molecule critical for leukocyte infiltration across the BBB [6]. These mediators play central roles in amplifying inflammatory cascades and promoting BBB disruption [4,5]. Beyond their role in maintaining barrier integrity, brain endothelial cells also serve as primary sensors of inflammatory stimuli, actively participating in immune surveillance and cytokine production at the neurovascular interface. The observation that both cell types respond to the extract at the same concentrations suggests consistent immunomodulatory activity across different cellular components of the neurovascular unit. These findings extend previous observations by Bottoni and collaborators [25], who reported a significant reduction in IL-6 in gastric epithelial cells treated with the same traditional aqueous extract at a concentration of 200 μg/mL [24,25]. Gastric epithelial cells, while primarily recognised for their barrier function, also participate actively in inflammatory responses through cytokine production and immune cell recruitment, particularly during infection or injury. The consistency of anti-inflammatory effects across peripheral and CNS cellular models supports the broad immunomodulatory activity of this traditional preparation, though the mechanisms underlying tissue-specific responses remain to be fully elucidated.

A notable finding of this study is the observation that *A. erba-rotta* subsp. *moschata* extract modulates the aryl hydrocarbon receptor (AhR) signalling pathway in brain endothelial cells. LPS stimulation suppressed AhR and its target gene CYP1A1 and expressions, whereas extract treatment restored their expression. The AhR pathway plays complex roles in immune regulation and inflammation [35,36]. While traditionally studied for xenobiotic metabolism [37], accumulating evidence indicates that AhR engagement can modulate immune cell function and cytokine production [38]. The induction of CYP1A1 suggests that phytochemical constituents of the extract may function as AhR ligands. Apigenin and luteolin, both identified as abundant constituents in our *A. erba-rotta* subsp. *moschata* extract [25], have been reported as AhR modulators [39,40] and are extensively documented for their ability to influence NF-κB signalling and reduce pro-inflammatory cytokine production in neuroinflammation models [13,14,15].

The restoration of AhR expression under LPS-induced suppression is noteworthy, as AhR downregulation has been associated with enhanced inflammatory responses [41,42]. However, definitive identification of the specific bioactive compounds responsible for AhR engagement and confirmation through ligand-binding assays or reporter gene systems would strengthen these findings.

BBB integrity represents a critical determinant of neuroinflammatory progression, as tight junction disruption allows peripheral immune cells and inflammatory mediators to infiltrate the CNS, thereby amplifying neuroinflammatory cascades [6,33,34]. Our results indicate that *A. erba-rotta* subsp. *moschata* extract influences the expression of key molecules associated with BBB integrity. In particular, the extract counteracted LPS-induced upregulation of matrix metallopeptidase-2 (MMP2), an enzyme known to degrade tight junction proteins [43,44], while preserving or enhancing the expression of tight junction components ZO-1 and occludin [45,46]. These effects are relevant given the role of BBB dysfunction in neurodegenerative diseases [6], where tight junction degradation allows peripheral immune cells and inflammatory mediators to infiltrate the CNS, exacerbating neuroinflammation [47,48]. Similar protective effects on BBB integrity have been reported for other natural compounds rich in flavonoids and polyphenols, such as resveratrol and quercetin [24,43]. Within the Achillea genus, *A. erba-rotta* subsp. *moschata* represents the first species to demonstrate such effects on tight junction-associated proteins in brain endothelial cells, combining modulation of MMP2 with concurrent support of ZO-1 and occludin expression. The mechanisms likely involve both the reduction in inflammatory cytokines known to influence BBB integrity [48] and potential direct interactions of phytochemicals with endothelial cells. However, causal relationships and the specific molecular pathways involved require validation through mechanistic studies using pathway inhibitors or genetic approaches.

Analysis of the Nrf2/HO-1 pathway in BV2 microglial cells revealed that *A. erba-rotta* subsp. *moschata* extract supports the cellular antioxidant response activated during inflammatory stress. LPS treatment significantly upregulated both Nrf2 and its downstream target HO-1, and co-treatment with the extract sustained these elevated levels. This represents a favourable characteristic, as the Nrf2 pathway constitutes a critical endogenous defence mechanism against oxidative stress [12]. These findings both corroborate and extend observations by Bottoni and collaborators [24], who reported Nrf2 activation in gastric epithelial cells. However, the extract appears to act differently depending on the cellular context: while strong basal activation was observed in epithelial cells, in microglia the extract did not significantly affect basal Nrf2 expression but instead maintained the response induced by inflammatory stimulation. It should be noted that these analyses were conducted at the mRNA level; protein-level validation, particularly assessment of Nrf2 nuclear translocation through Western blot or immunofluorescence, would provide more definitive evidence of pathway activation. The ability of the extract to maintain HO-1 expression while simultaneously reducing pro-inflammatory mediators suggests a balanced immunomodulatory profile that supports cellular defence mechanisms.

Treatment with *A. erba-rotta* subsp. *moschata* induced upregulation of transforming growth factor-β (TGF-β) in LPS-stimulated BV2 cells, suggesting a shift toward anti-inflammatory and reparative signalling. TGF-β plays crucial roles in resolving inflammation and promoting tissue homeostasis [49,50], potentially through microglial polarisation toward a reparative phenotype [51]. This finding suggests that *A. erba-rotta* subsp. *moschata* may not only attenuate acute inflammatory responses but also facilitate the transition to resolution and repair phases. To our knowledge, such modulation of TGF-β has not been reported for other Achillea species in microglial models. However, the functional consequences of this upregulation and its relationship to microglial polarisation states require further investigation through phenotypic marker analysis and functional assays.

Within the Achillea genus, several species have demonstrated anti-inflammatory and antioxidant properties. Elmann and collaborators [18] reported that A. fragrantissima extract reduced neuroinflammatory markers in microglial cultures through modulation of NO production and cytokine release. In line with these findings, *A. erba-rotta* subsp. *moschata* exhibits similar anti-inflammatory activity in the microglial model. While both species effectively target NO production via iNOS downregulation, *A. erba-rotta* subsp. *moschata* demonstrates effects on multiple inflammatory mediators (IL-6, TNF-α, iNOS) and additionally modulates TGF-β expression. Additionally, *A. millefolium* and *A. ligustica* have demonstrated anti-inflammatory properties in peripheral models [18]. Our study extends these observation to both microglial and endothelial components of the neurovascular unit, revealing effects on BBB integrity markers not previously reported for other Achillea species. However, direct quantitative comparisons between species should be interpreted cautiously due to differences in extraction methods, cellular models, and experimental conditions. The observed biological activities can be attributed to the phytochemical composition of *A. erba-rotta* subsp. *moschata*, particularly its flavonoid content. Previous characterisation has identified glucosides and aglycones of quercetin, apigenin, and luteolin, with apigenin-7-malonyl glucoside showing remarkable relative abundance in the traditional preparation [25]. The multi-target effects observed in our study are consistent with the well-documented pleiotropic properties of these compounds [10,11,12], though the specific contributions of individual phytochemicals remain to be definitively established.

Despite these findings, several mechanistic limitations should be acknowledged. First, while we observed modulation of inflammatory mediators (IL-6, IL-1β, TNF-α, iNOS), we did not directly assess NF-κB phosphorylation status or nuclear translocation. The reduction in downstream inflammatory mediators suggests involvement of NF-κB-dependent pathways, but causal relationships remain to be established through pathway inhibition studies or assessment of NF-κB activation states. Second, modulation of the Nrf2/HO-1 pathway was assessed at the mRNA level. While transcriptional changes generally correlate with protein expression, confirmation through protein-level analysis, particularly assessment of Nrf2 nuclear translocation, would provide more definitive evidence of pathway activation. Third, AhR pathway engagement was inferred from CYP1A1 induction and AhR expression changes. While this provides evidence of pathway modulation, direct demonstration through ligand-binding assays, reporter gene systems, or assessment of AhR nuclear translocation would strengthen mechanistic conclusions. The identification of specific phytochemical constituents responsible for AhR modulation remains an important area for future investigation. Finally, the complex phytochemical composition of the extract limits our ability to attribute specific effects to individual compounds. While apigenin, luteolin, and quercetin are likely contributors based on their known properties and abundance in the extract, isolation studies and structure–activity relationship analyses would be required to definitively identify the bioactive components responsible for each observed effect.

**Figure 8:**
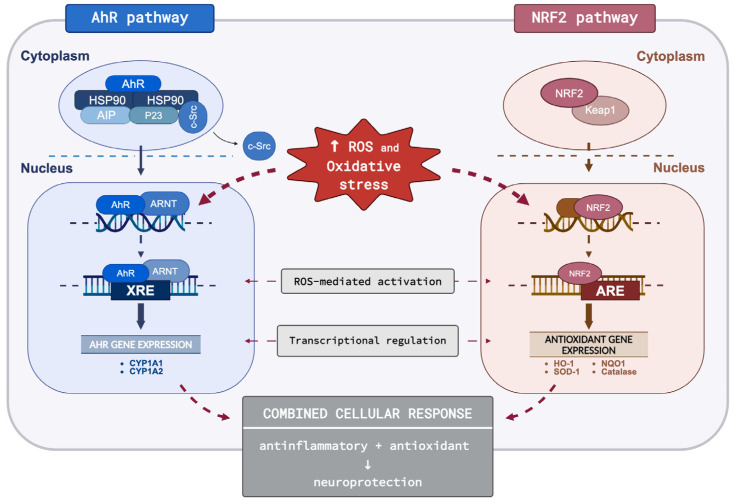
Crosstalk between AhR and Nrf2 pathways in the regulation of inflammation and oxidative stress. Schematic representation of AhR and Nrf2 signalling pathways and their convergent activation in response to oxidative stress. Upon ROS generation, AhR translocates to the nucleus and binds to xenobiotic response elements (XRE), inducing expression of detoxification enzymes. Concurrently, Nrf2 translocates to the nucleus and binds to antioxidant response elements (ARE), activating transcription of antioxidant genes. The pathways exhibit ROS-mediated signalling (dashed arrows), converging to produce a combined cellular response characterised by integrated anti-inflammatory and antioxidant effects that result in neuroprotection. This dual pathway activation represents a central mechanism of action for *A. erba-rotta* subsp. *moschata* phytochemicals in brain cell models. Created in BioRender. Mercuriali, B. (2026); https://app.biorender.com/illustrations/695a8cdd335332a3149ae2ca accessed on 13 April 2026.

Overall, these results collectively indicate that *A. erba-rotta* subsp. *moschata* modulates interconnected inflammatory and antioxidant pathways in brain cell models (Figure 8). The concomitant maintenance of HO-1 expression and reduction in pro-inflammatory mediators, coupled with effects on BBB integrity markers and AhR-CYP1A1 signalling, suggest coordinated immunomodulatory activity that warrants further mechanistic investigation and in vivo validation.

### Study Limitations and Future Directions

While this study provides valuable mechanistic insights, several limitations should be acknowledged and addressed in future research. First, our findings are based on in vitro immortalised cell lines; validation in primary cells and animal models of neuroinflammation is essential. Second, key pathways were assessed primarily at the mRNA level; protein-level confirmation would strengthen these findings. Third, we demonstrate associations but not causality; studies using pathway inhibitors would confirm direct mechanistic involvement. Fourth, pharmacokinetic parameters including bioavailability and blood–brain barrier penetration remain unknown. Fifth, natural extracts may show batch-to-batch variability; standardisation protocols are needed. Future studies addressing these limitations through in vivo validation, mechanistic confirmation, and pharmacokinetic characterisation will be essential to establish therapeutic relevance.

## 4. Material and Methods

### 4.1. Plant Material

For pharmacological analysis, the flower heads of *A. erba-rotta* subsp. *moschata* were collected in Valmalenco (Alpe Mastabbia—2200 m a.s.l., Chiesa in Valmalenco, Sondrio, Lombardy, Italy) in July 2022, during an ethnobotanical field investigation and with the support of key informants. Prof. G. Fico and Prof. C. Giuliani identified the species according to Pignatti et al. [20] and voucher specimens were deposited in the Herbarium of the Ghirardi Botanical Garden of the University of Milan (DISFARM, Toscolano Maderno, Brescia, Italy), with the identification codes GBG123/GBG124.

### 4.2. Preparation of the Extract

The plant material was air-dried and stored away from light and heat sources before the extraction. An aqueous extract (traditional decoction) was prepared, based on ethnobotanical primary data [25,26]. To prepare the decoction, 2 g of dried plant material was added to 100 mL of deionised water. After reaching boiling point, the heat was turned off, and the mixture was left to infuse for 20 min before being filtered through Whatman filter paper. The resulting aqueous extract was freeze-dried overnight. The lyophilised extract was reconstituted in a 1:1 (*v/v*) solution of H_2_O and DMSO to a final concentration of 25 mg/mL. All aliquots were stored at −20 °C until use.

### 4.3. Phytochemical Characterisation

The phytochemical profile of *A. erba-rotta* subsp. *moschata* aqueous extract was previously characterised using LC-MS/MS, according to the procedure reported in [24]. Major constituents include chlorogenic acid derivatives, flavones (apigenin, luteolin), and flavonols (quercetin, kaempferol, isorhamnetin). The traditional decoction preparation method was used to ensure consistency with characterised material. Identified compounds’ concentrations were determined by integration of HPLC-PDA profile using gallic acid as internal standard and reported as µg of gallic acid equivalents per mL of extract solution (see Supplementary data, Table S1).

### 4.4. Cell Culture

Immortalised human brain microvascular endothelial cells (HBMECs) (P10361-IM—Innoprot, Bizkaia, Spain) were grown on flasks coated with 50 μg mL^−1^ fibronectin (Sigma Aldrich, Darmstadt, Germany) in MCDB 131 culture medium (Gibco, Thermo Fisher Scientific, Waltham, MA, USA—1.0 g/L D-glucose and 2 mM L-glutamine) supplemented with 5% fetal bovine serum (FBS) 1% endothelial cell growth supplement (ECGS) (Sigma—E2759), 0.1% hydrocortisone (Sigma—H0396) and antibiotics (100 U/mL penicillin and 100 µg/mL streptomycin). Cells between passages 12 and 14 were used and incubated at 37 °C, 5% CO_2_, in a humidified atmosphere.

Murine microglial cells (BV-2) were maintained in culture medium (DMEM, Dulbecco’s Modified Eagle Medium, GlutaMAXTM, 4.5 g/L D-glucose, 25 mM Hepes and 15 mg/L Phenol red) supplemented with 10% FBS and antibiotics (100 U/mL penicillin and 100 µg/mL streptomycin). Cells were maintained at 37 °C in a humidified environment with 5% CO_2_.

### 4.5. Cell Treatments

Human brain microvascular endothelial cells (hBMECs) were seeded in a 6-well plate at a density of 6.0 × 10^4^ cells per well; after 36 h, when cells confluence reached approximately 70%, the medium was replaced with serum-free MCDB-131 (1.0 g/L D-glucose, 2 mM L-glutamine, 1% of penicillin/streptomycin) to induce starvation conditions for an overnight period. The following day, cells were stimulated with lipopolysaccharide from *E. coli* (LPS—Sigma Aldrich, Germany) (200 µg/mL) and treated with *A. erba-rotta* subsp. *moschata* extract (20 and 200 µg/mL) for 6 h.

**Figure 9:**
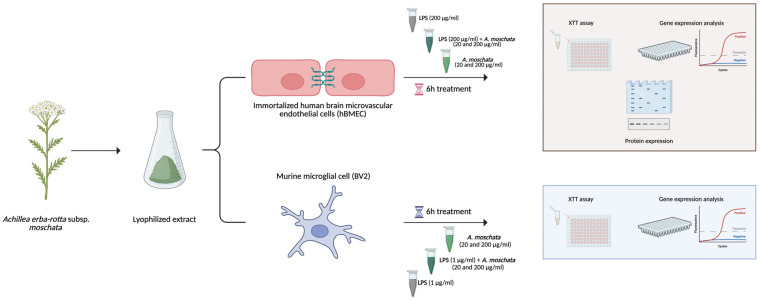
Experimental design of *Achillea erba-rotta* subsp. *moschata* treatments in brain-related cellular models. A schematic overview of the experimental protocol used to investigate the effects of the aqueous extract of *A. erba-rotta* subsp. *moschata* in microglial BV2 cells and human brain microvascular endothelial cells (hBMECs). Cells were treated with the extract (20 or 200 µg/mL) with lipopolysaccharide (LPS) for 6 h, followed by the assessment of cell viability, inflammatory mediators and antioxidant pathways. The figure summarises treatment timing and the experimental workflow applied throughout the study. Created in BioRender. Mercuriali, B. (2026); https://app.biorender.com/illustrations/693436258102c8de7a7e8c51?slideId=8fc4ad88-c227-4565-94e9-bc4254f477b1 accessed on 13 April 2026.

Murine microglial BV-2 cells were seeded in a 12-well plate at a density of 8.0 × 10^4^ cells per well; after 36 h, when cells confluence reached approximately 70%, the medium was replaced with serum-free medium (containing the same basal components and 1% penicillin/streptomycin) to induce a starvation condition overnight. The following day, cells were stimulated with LPS (1 µg/mL) and treated with *A. erba-rotta* subsp. *moschata* extract (20 and 200 µg/mL) for 6 h. All experiments were carried out in three independent replicates (Figure 9).

The extract was diluted from a stock solution (25 mg/mL in 1:1 H_2_O:DMSO) in culture medium. All experimental groups, including controls, received equivalent DMSO concentrations to normalise solvent effects.

The tested concentrations (20 and 200 µg/mL) were selected based on previous studies demonstrating significant anti-inflammatory activity of *A. erba-rotta* subsp. *moschata* extracts in this range, with no cytotoxicity up to 200 µg/mL [24,25]. The lower concentration (20 µg/mL) also represents a physiologically plausible exposure, estimated from traditional preparation parameters (3–5 g dried flowers in 150–200 mL water) assuming ~10% oral bioavailability and distribution in ~5 L blood volume.

### 4.6. RNA Isolation and Quantitative RT-PCR

Total RNA was extracted from cells cultured in 6- and 12-well plates using the Direct-zol RNA Microprep kit (Zymo Research, Irvine, CA, USA), following the manufacturer’s protocol. RNA concentrations were determined using a GE Healthcare NanoVue Plus UV–Vis spectrophotometer. Subsequently, 1 μg of total RNA was reverse transcribed into cDNA using the SensiFAST™ cDNA Synthesis Kit (Meridian Bioscience, Cincinnati, OH, USA). Quantitative PCR was performed with SensiFAST SYBR No-ROX kit (Meridian Bioscience, Cincinnati, OH, USA) on a Bio-Rad CFX Connect Real-Time PCR System. All experiments were conducted in triplicate and gene expression levels were calculated using the 2^−ΔΔCt^ method and normalised to the mean expression of the RPL13a housekeeping gene. Primer sequences are listed in Table 1A,B. PCR cycling conditions included an initial denaturation at 95 °C for 3 min, followed by 39 cycles of 95 °C for 10 s and 60 °C for 30 s.

### 4.7. Western Blot Analysis

Proteins were extracted from hBMECs with RIPA lysis buffer (150 mM NaCl, 10 mM, Tris, pH 7.4, 5 mM EDTA, 0.1% SDS, 1% sodium deoxycholate and 1% Triton X-100) added with protease inhibitors (Roche Diagnostics, Mannheim, Germany) and phosphatase inhibitors (Sigma-Aldrich, Germany) according to manufacturer’s instructions. The lysates were sonicated and subsequently spun at 13.000 rpm for 15 min to isolate the whole cell lysate; supernatants were collected and assayed for protein quantification by Bradford method [52]. Aliquots of the protein extracts were separated into single-use samples and stored at −80 °C until use. An aliquot of 20–30 µg of total protein was loaded into an SDS-polyacrylamide gel and then transferred to nitrocellulose membranes using the iBlot 2 Dry Blotting System (Thermo Fisher Scientific, USA). Membranes were reversibly stained with Ponceau S, to verify transfer efficiency, and subsequently blocked for 1 h at room temperature in 5% non-fat dry milk in TBS-T (20 mM Tris-HCl pH 7.6, 150 mM NaCl, 0.1% Tween-20). The membranes were then incubated overnight at 4 °C with specific primary antibodies (Table 2), under gentle agitation.

After washing in TBS-T, membranes were incubated with goat anti-mouse HRP-labelled secondary antibody (Bio-Rad Laboratories, San Francisco, CA, USA) (1:2500 in 5% non-fat dry milk in TBS-TWEEN 20 0.1%) or with goat anti-rabbit HRP-labelled secondary antibody (Sigma Aldrich, Germany) (1:2500 in 5% non-fat dry milk in TBS-TWEEN 20 0.1%) at room temperature for 2 h. To enable detection of proteins with varying molecular weights, membranes were horizontally cut as needed. For blots requiring sequential detection with multiple antibodies, membranes were stripped using a buffer composed of 100 mM 2-mercaptoethanol, 2% SDS, and 62.5 mM Tris-HCl (pH 6.7) for 45 min at 50 °C, followed by re-incubation with additional primary and secondary antibodies. Signal detection was performed using ECL reagent (Cyanagen, Bologna, Italy), and bands were quantified via densitometry using a ChemiDoc Imaging System (Bio-Rad) and ImageJ software (version 1.54g). All target proteins were normalised to β-actin expression from the same membrane. Normalised values are expressed as fold change relative to control conditions. Each Western blot experiment was performed with three independent biological replicates, and representative blots are shown in figures.

### 4.8. Cell Viability (XTT)

Cell viability was determined using the XTT assay, a colorimetric method that evaluates mitochondrial metabolic activity as an indirect measure of viable cell number. The assay is based on the reduction in the tetrazolium salt XTT (2,3-Bis-(2-methoxy-4-nitro-5-sulfophenyl)-2H-tetrazolium-5-carboxanilide) to a soluble orange formazan product by mitochondrial enzymes in metabolically active cells. After treatment, cells were incubated with the XTT labelling mixture (Cell Proliferation Kit II, Roche) in culture medium, according to the manufacturer’s instructions, and maintained at 37 °C in a humidified atmosphere containing 5% CO_2_ for 2 h. Absorbance was then measured at 450 nm with a reference wavelength of 630 nm using a microplate reader. Cell viability was expressed as a percentage relative to untreated control cells, considered 100% viable.

### 4.9. Statistical Analysis

All data are presented as mean ± standard deviation (SD) from at least three independent experiments. Prior to parametric testing, data normality was assessed using the Shapiro–Wilk test and homogeneity of variance was evaluated using Levene’s test. Statistical significance was determined by one-way ANOVA followed by Sidak’s multiple comparison post hoc test. *p*-values < 0.05 were considered statistically significant, with standard notation: * *p* < 0.05, ** *p* < 0.01, *** *p* < 0.001, **** *p* < 0.0001. All statistical analyses were performed using GraphPad Prism 9 (GraphPad Software, San Diego, CA, USA).

## 5. Conclusions

This study provides the first comprehensive characterisation of *A. erba-rotta* subsp. *moschata* neuroprotective activities in brain cell models. Our findings demonstrate multi-target modulation of neuroinflammatory pathways, including reduction in pro-inflammatory mediators in both microglial and endothelial cells, preservation of blood–brain barrier tight junction integrity, engagement of the AhR-CYP1A1 signalling axis, and support of endogenous antioxidant defences. In addition, the upregulation of TGF-β suggests facilitation of inflammatory resolution and tissue homeostasis. These findings provide mechanistic support for the traditional use of this Alpine medicinal plant and suggest its potential value as a source of natural neuroprotective compounds for further investigation in neurodegenerative disease contexts.

## Figures and Tables

**Table 1:** List of human (**A**) and mouse primers (**B**) used for real-time PCR analysis.

(**A**)		
**Primer**	**Sequence Forward (3′-5′)**	**Sequence Reverse (5′-3′)**
IL-6	GGTACATCCTCGACGGCATCT	GTGCCTCTTTGCTGCTTTCAC
IL-1β	CACGATGCACCTGTACGATCA	GTTGCTCCATATCCTGTCCCT
AhR	CAGTACTGCCAGGCCAACA	TGTGTGGTAGTCTGAGTGTTATTTATG
CYP1A1	GCTGACTTCATCCCTATTCTTCG	TTTTGTAGTGCTCCTTGACCATCT
ICAM-1	TGTGACCAGCCCAAGTTGTT	AGTCCAGTACACGGTGAGG
ZO-1	TATTATGGCACATCAGCACG	TGGGCAAACAGACCAAGC
RPL13α	GGATGAACACCAACCCTTCC	AACACCTTGAGACGGTCCAG
(**B**)		
IL-6	CAAAGCCAGAGTCCTTCAGA	GCCACTCCTTCTGTGACTCC
TNF-α	GCCTCTTCTCATTCCTGCTT	AGGGTCTGGGCCATAGAACT
iNOS	ACCAAGCTGAACTTGAGCGA	GCCCCATAGGAAAAGACTGC
Nrf2	ACAGTGCTCCTATGCGTGAA	GAGCCTCTAAGCGGCTTGAA
HO-1	TGCTAGCCTGGTGCAAGATA	GCCAACAGGAAGCTGGAGAGT
RPL13α	ACAGCCACTCTGGAGGAGAA	GAGTCCGTTGGTCTTGAGGA

**Table 2:** List of primary antibodies used for Western blot analysis.

Antigen	Host	Dilution	Polyacrylamide Gel	Company	Catalogue Number
Occludin	rabbit	1:1000	10%	Cell Signalling (Danvers, MA, USA)	91131
AhR	rabbit	1:500	10%	Novus Biologicals, (Englewood, CO, USA)	NB100-2289
β-actin	mouse	1:500	10%	Sigma Aldrich (Germany)	A5441

## Data Availability

The original contributions presented in the study are included in the article, further inquiries can be directed to the corresponding author.

## Supplementary Materials

The following supporting information can be downloaded at: https://www.mdpi.com/article/10.3390/ph19060832/s1.
