# Supplementary material for: Achillea erba-rotta subsp. moschata (Wulfen) I. Richardson Modulates Inflammatory and Antioxidant Pathways in Brain Endothelial and Microglial Cells
Source: Pharmaceuticals (Basel). 2026 May 27;19(6):832. doi: 10.3390/ph19060832 (PMC13304942; doi:10.3390/ph19060832)
Supplement: Supplementary file 1 [file pharmaceuticals-19-00832-s001.zip › pharmaceuticals-4287384-supplementary.pdf]

Supplementary data

Table S1. Concentrations of identified compounds in phytochemical profile of Achillea erba-rotta subsp. moschata aqueous extract; concentrations are expressed as µg of gallic acid equivalents per mL of extract solution.

#	**Compound name**	**Q.ty (µgGAE/mL)**
1	Caffeoylquinic acid isomer 1	0.308
2	Caffeoylquinic acid isomer 2	12.038
3	Vicenin-2	0.619
5	Schaftoside	0.116
6	Quercetin-3O-rutinoside (Rutin)	0.541
7	Kaemferol-O-glucoside	0.514
8+9	Luteolin-O-glucoside +Mearnsetin hexoside isomer 2	0.316
11	Dicaffeoylquinic acid isomer 1	0.218
12	Isorhamnetin-O-hexoside isomer 1	0.007
13	Dicaffeoylquinic acid isomer 2	9.667
14	Isorhamnetin-O-rutinoside	0.026
15+16	Apigenin-7-glucoside + Isorhamnetin-O-hexoside isomer 2	2.948
17+18	Syringetin-3O-glucoside + Isorientin-7-methylester	2.458
19	Dicaffeoylquinic acid isomer 3	0.045
20	Luteolin-7-malonyl-glucoside	0.025
21	Eupatolin	0.225
22	Apigenin-7-malonyl-glucoside	0.205
25	Apigenin	3.318
23	Luteolin	0.043
24	Axillarin	0.046
26	Chrysoeriol/Hispidulin	0.033
29-30	Quercetin-3,3'-dimethylether + 6-hydroxykaempferol-3,6-dimethylether	0.108
31	Chrysosplenol-D/Jaceidin	0.272
